# Text, talk, screen: mapping mHealth interventions for cervical cancer prevention and control in Africa — a scoping review

**DOI:** 10.3389/fdgth.2026.1947988

**Published:** 2026-10-15

**Authors:** Alexis Harerimana, Gugu Mchunu

**Affiliations:** 1 Faculty of Health Sciences, Durban University of Technology, Durban, KwaZulu-Natal, South Africa; 2 College of Healthcare Sciences, James Cook University, Townsville, QLD, Australia

**Keywords:** Africa, cervical cancer, cervical cancer control, cervical cancer prevention, cervical cancer screening, health intervention, mHealth, mobile health

## Abstract

**Background:**

Cervical cancer continues to be a major cause of morbidity and mortality among women in Africa. Screening coverage across the continent remains significantly below the World Health Organization's 90–70–90 targets. Mobile health (mHealth) technologies offer a scalable solution to improve outreach, screening, vaccination, and follow-up in resource-limited settings. Thus, this scoping review aims to systematically map the existing evidence on mHealth interventions for the prevention and control of cervical cancer in Africa.

**Methods:**

This scoping review adopted the methodological framework developed by Arksey and O'Malley and refined by Levac et al. A systematic search was conducted across CINAHL, MEDLINE, Scopus, and Web of Science, complemented by manual searches. Studies published in English between 2016 and 2026 were eligible. From an initial set of 550 records, 32 studies met the inclusion criteria and underwent subsequent data extraction and thematic analysis.

**Findings:**

mHealth interventions included messaging, voice communication, and awareness-raising (*n* = 14), image-based diagnosis with tele-expert review (*n* = 11), digital apps for decision-making and support (*n* = 3), baseline and feasibility studies (*n* = 3), and provider education and training (*n* = 5). Stages of the cervical cancer care continuum were not mutually exclusive: studies addressed screening (*n* = 22), diagnosis and treatment (*n* = 9), primary prevention/HPV vaccination (*n* = 3), post-treatment follow-up (*n* = 1), and workforce and system-capacity interventions (*n* = 2). Electronic devices used included smartphones (*n* = 12), basic mobile phones (*n* = 13), computer- and cloud-based devices (*n* = 3), a digital camera (*n* = 1), a tablet (*n* = 1), and unspecified mobile phones (*n* = 2). Outcomes included diagnostic accuracy, feasibility and acceptability, treatment uptake, provider competence, and vaccination uptake. Facilitators included high mobile phone ownership and real-time expert support, while challenges involved sustainability, health systems, connectivity, privacy and confidentiality concerns, low literacy, and language barriers.

**Conclusion:**

mHealth shows promise for cervical cancer prevention and control in Africa. Realizing this potential at scale requires investing in underrepresented care stages, addressing connectivity and health-system barriers, and conducting rigorous multi-country research beyond pilot evidence.

## 1 Background

Cervical cancer is a formidable global health challenge, with an estimated 662,044 new cases and 348,709 deaths in 2022, making it the fourth leading cause of cancer incidence and mortality among women worldwide (1). This burden is disproportionately borne by Africa, which accounted for approximately 125,699 new cases and 80,614 deaths in 2022, representing 19% of global incidence and 23% of global mortality, despite a comparatively smaller share of the global population (2). Nearly 90% of these cases occur in contexts characterized by low and medium Human Development Index scores (2, 3). In response to this global burden, the World Health Organization launched the ‘Global Strategy to Accelerate the Elimination of Cervical Cancer as a Public Health Problem’, establishing 90–70–90 targets for human papillomavirus (HPV) vaccination coverage, screening coverage, and treatment access by 2030 (4). However, cervical cancer screening coverage remains highly unequal: only 35% of women in low-income countries and 55% in lower-middle-income countries (LMICs) have been screened, compared with 80% in high-income countries (HICs) (4, 5).

mHealth plays a vital role in the prevention and management of cervical cancer, as the process from identifying risk factors to implementing preventive measures involves a sequence of repeated actions, including outreach, screening participation, vaccination, communication of results, and subsequent follow-up (6–8). Studies from HICs highlighted the importance of mHealth in cervical cancer screening. A population-based trial in Portugal demonstrated that automated text messages and phone calls boosted screening adherence from 25.7% to 39.0%, suggesting that low-cost reminders can alter behavior when screening services are accessible (9). In Sweden, Broberg et al. (10) evaluated an intervention comparing the standard cervical cancer screening invitation routine alone with the standard routine supplemented by a telephone call; screening rates in the intervention group reached 13.0% compared to 3.9% in the control group at three months, and 18.0% compared to 10.6% at twelve months. Similarly, in the USA, Ganta et al. (11) documented an increase from a baseline of 2.47% to 11.8% following the timely reminder intervention via sequential text messages and phone calls. A pragmatic trial in London found that behavioral SMS messages increased participation in cervical cancer screening (12). In Australia, a randomized trial involving Aboriginal and Torres Strait Islander women showed that phone/SMS reminders were at least as effective as letters and more cost-efficient, although the difference between groups was not statistically significant (13). Similarly, in Malaysia, most women received SMS results without technical issues and expressed satisfaction with the system (14). An ATICA study (Application of Communication and Information Technologies for Self-Collection; acronym in Spanish) found high acceptability of SMS for triage follow-up (15). Likewise, a brief mHealth intervention in southern India improved knowledge of cervical cancer and modestly increased Pap smear uptake (16).

In LMICs, particularly in Africa, mHealth is vital for primary prevention of cervical cancer through HPV vaccination. Researchers and health systems across the continent have piloted a diverse array of mHealth applications, including one-way and two-way SMS or audio reminders and educational messaging (17–19), smartphone-based cervical image capture augmented by remote expert review (20–22), and dedicated provider- and patient-facing applications (23, 24). Furthermore, videoconferencing-based tele-mentoring platforms (25) and artificial intelligence-supported diagnostics are used in mHealth in Africa (26). Findings from studies conducted in Africa demonstrate the importance of mHealth in cervical cancer prevention and control. In Uganda, automated text and phone reminders improved timely vaccination (27), and randomized trials in Nigeria and Kenya reported higher HPV vaccine uptake among enrolled adolescent girls and their guardians who received mHealth messaging (28, 29). In Nigeria, mHealth increased Pap smear uptake compared to standard care (19), and Ogunsola et al. (30) documented an increase in visual inspection with acetic acid (VIA)-positive concordance from 26% to 42% across successive phases of a smartphone-supported screening program involving 29,262 women.

In African countries, mHealth technologies are increasingly used to overcome barriers to cervical cancer screening and treatment. In Ghana, culturally adapted WhatsApp audio messages in Fante/Akan were used to deliver screening education to women living with HIV (18); in Kenya, smartphone-based cervical imaging with remote expert review was used to support screening among women living with HIV (31); in Eswatini, cervical images captured on smartphones were transmitted to a remote expert to support VIA screening (22); in Uganda, nurses used a smartphone-based telemedicine system for case review in cervical cancer screening (32); in Cameroon, Project ECHO videoconferencing was used to tele-mentor providers in cervical cancer prevention and treatment (25); and in Rwanda, national digital registries integrated with the OpenMRS platform supported the tracking of HPV vaccination, screening, and treatment (24). Studies from Zambia and the Democratic Republic of the Congo demonstrate how smartphone imaging and artificial intelligence (AI) are enhancing visual cervical screening (33–35). In Kinshasa, Democratic Republic of the Congo, Tendobi et al. (33) trained non-specialist providers to conduct combined acetic acid and Lugol's iodine inspections using smartphone cervicography and remote expert review. Among 480 women, this method achieved 66.0% sensitivity and 87.8% specificity for any cytological lesion, with a negative predictive value of 99.7% for high-grade lesions (33). In Lusaka, Zambia, Parham et al. (34) developed a 12-level risk scale by combining HPV genotyping with AI-based automated visual evaluation (AVE) of smartphone images. This scale accurately predicted histologically confirmed precancer, even among women living with HIV. Furthermore, a study by Hu et al. (35) reported AVE sensitivity of 85% and specificity of 86% (AUC 0.91) involving 8,204 Zambian women.

Although mHealth technologies show promise for cervical cancer prevention and control, evidence of effectiveness is mixed and implementation barriers have been reported. For instance, in Ghana, a culturally tailored SMS program did not increase screening attendance (17), and studies commonly report connectivity failures, sustainability concerns, and workforce or resource constraints as obstacles across intervention types (31, 32). Despite this proliferation of primary research on mHealth, the existing evidence base remains fragmented. Thus, this review aims to systematically map the existing evidence on mHealth interventions for the prevention and control of cervical cancer in Africa.

## 2 Methods

This scoping review followed the methodological framework for scoping reviews developed by Arksey and O'Malley (36) and refined by Levac et al. (37). This framework consists of five core stages: identifying the research question, identifying relevant studies, selecting studies, charting the data, and collating, summarizing, and reporting the results. Arksey and O'Malley (36) also proposed an optional sixth stage, stakeholder consultation. We did not undertake a consultation exercise; therefore, the findings reflect only the published literature and have not been contextualized by stakeholder perspectives. The review was reported in accordance with the Preferred Reporting Items for Systematic Reviews and Meta-Analyses Extension for Scoping Reviews (PRISMA-ScR) (38).

### 2.1 Stage 1: identifying the research question

The review was guided by the following research question: “What is the nature and extent of mHealth interventions targeting cervical cancer prevention and control in Africa?” The following are the sub-questions:

- What types of mHealth interventions have been implemented for cervical cancer prevention and control in Africa?
- Which stages of the cervical cancer care continuum have mHealth interventions targeted in Africa?
- What outcomes have been reported for mHealth interventions targeting cervical cancer in Africa?
- What facilitators have been reported for mHealth interventions targeting cervical cancer in Africa?
- What are the challenges for mHealth interventions targeting cervical cancer in Africa?

### 2.2 Stage 2: identifying relevant studies

A systematic search was conducted across the following databases: CINAHL, MEDLINE, Scopus, and Web of Science. This search was supplemented by manual searches using Google and Google Scholar. The search strategy comprised the following search terms combined using Boolean operators: mHealth, mobile health interventions, cervical cancer prevention and control, and Africa. Subject headings were exploded where applicable (denoted by “exp” in MEDLINE and by the “+” symbol in CINAHL), so that all narrower terms were retrieved together with the broader heading. This included the regional descriptor “Africa”, which captured its sub-regions and individual countries without the need to list each country separately. This approach ensured comprehensive and consistent coverage, minimized the risk of inadvertently omitting or misspelling countries in an extensive search strategy, and improved the reproducibility and manageability of the search process. In contrast, in Scopus and Web of Science, which do not have a hierarchical controlled vocabulary, subject headings could not be exploded, so the same concepts were searched as free-text terms in the title, abstract, and keyword fields (TITLE-ABS-KEY in Scopus and Topic search in Web of Science). The regional descriptor “Africa” was searched as a free-text term (Africa* or African*) without listing individual countries, so records that name only a specific country may not have been retrieved in these two databases. This has been acknowledged as a limitation and recommendations for future studies were made to use the suggested approach by Pienaar et al. (39).

We used Google and Google Scholar to identify gray literature and unindexed records, including preprints. Neither platform supports controlled vocabularies or term expansion, and Google Scholar further limits query length. Therefore, simplified keyword combinations of the main concepts with “Africa” or “African” were employed, rather than the full database strategy or extensive lists of country names. Because results are ranked by relevance and cannot be exported comprehensively, the first 100 results from each Google Scholar search and the first 10 pages from each Google search were screened. This approach is commonly used to make gray literature searching feasible, as suggested by Haddaway et al. (40).

The search strategy integrated four conceptual blocks, each addressing a specific aspect of the review question. Block S1 focused on the intervention or delivery mode, including mobile, digital, and telehealth technologies. **Block S2** covered the health condition: cervical cancer and its causative agent, human papillomavirus. **Block S3** encompassed cervical cancer prevention activities of interest, including screening, testing, vaccination, and related prevention and control approaches (e.g., Pap smear, VIA, HPV testing, HPV vaccination). Finally, Block S4 defined the geographical context: Africa. Terms within each block were combined using “OR” to capture synonyms and variant spellings. The four blocks were then linked with “AND” (S5 = S1 AND S2 AND S3 AND S4) to ensure that only records addressing all four concepts were retrieved.

Blocks S2 and S3 were deliberately combined using “AND” rather than “OR” because they are complementary. S2 alone would yield the entire cervical cancer literature, including topics such as treatment, staging, survival, and epidemiology, which are beyond this review's scope. S3 alone would retrieve screening, testing, and vaccination studies for other conditions and would capture ambiguous terms such as “VIA” and “HPV testing” that are used outside the cervical cancer context. Combining S2 and S3 with “AND” ensured that only records addressing both cervical cancer and a relevant prevention or screening activity were retained. This approach balances sensitivity and precision, maintaining a manageable number of records for screening. We recognize that this approach might exclude records that mention a prevention/screening activity without using cervical-cancer-specific terminology (or vice versa), and this has been added in the Limitations and Recommendations section. Searches were conducted across the selected electronic databases between April and June 2026. This timeframe allowed for the identification of relevant literature indexed or published during the search process, ensuring the review captured the most current evidence available at the time of study selection. Search strings were customized to databases, and below is an example of a CINAHL/EBSCOhost-syntax search string for each block:

#### 2.2.1 Block S1: intervention or delivery mode (digital and mobile health)

**S1.** (MH “Telehealth+” OR MH “Cell Phones+” OR MH “Text Messaging+” OR MH “Mobile Applications+”) OR TX (mHealth OR “mobile health” OR “mobile phone*” OR cellphone* OR “cell phone*” OR smartphone* OR “text messag*” OR SMS OR “short message service” OR “mobile application*” OR “mobile app” OR “mobile apps” OR WhatsApp OR “digital health” OR eHealth OR telehealth OR telemedicine OR “mobile technolog*” OR “digital intervention*”).

#### 2.2.2 Block S2: health condition (cervical cancer and HPV)

**S2.** (MH “Cervix Neoplasms+” OR MH “Papillomavirus Infections+”) OR TX (“cervical cancer*” OR “cervical neoplasm*” OR “uterine cervical neoplasm*” OR “cancer* cervix” OR “cervical carcinoma*” OR HPV OR “human papillomavirus*”)

#### 2.2.3 Block S3: prevention, screening and control activities

**S3.** (MH “Early Detection of Cancer+” OR MH “Mass Screening+” OR MH “Papanicolaou Test+” OR MH “Papillomavirus Vaccines+”) OR TX (“cervical screening” OR “cervical cancer screening” OR “Pap smear*” OR “Papanicolaou test*” OR “visual inspection with acetic acid” OR VIA OR “HPV test*” OR “HPV vaccin*” OR “cervical cancer prevention” OR “cervical cancer control”)

#### 2.2.4 Block S4: geographical context (Africa)

**S4.** MH “Africa+” OR TX (Africa* OR African*)

#### 2.2.5 Block S5: combined search

**S5.** S1 AND S2 AND S3 AND S4

### 2.3 Stage 3: selecting relevant studies

Study selection proceeded via a two-stage, iterative process consistent with the approach recommended by Levac et al. (37). Two reviewers independently screened a calibration sample of titles and abstracts against preliminary eligibility criteria. Discrepancies were then discussed, and the criteria refined accordingly. The remaining records were subsequently screened independently by two reviewers. The full texts of potentially eligible studies were independently assessed against the refined criteria, and reasons for exclusion were documented. Eligible studies included those of any design that described an mHealth intervention or mHealth readiness for cervical cancer prevention and control among women or females in an African setting and were published in English between 2016 and 2026. This timeframe was chosen because it coincides with the rapid expansion of mobile-phone and smartphone access across Africa and with the rise of cervical cancer prevention on the global and continental public health agenda, notably the WHO global strategy for cervical cancer elimination and its 2030 90–70–90 targets (6) and the wider roll-out of HPV vaccination. It also allowed the review to focus on contemporary, Africa-specific evidence, since earlier syntheses of digital health for cervical cancer were global or low- and middle-income-country-wide in scope rather than Africa-specific (6–8). To comprehensively map the available evidence, including emerging and gray literature, as is the aim of scoping reviews (36, 37), we included preprints alongside peer-reviewed publications. Preprints offer the most recent findings before formal publication, which is particularly valuable in rapidly evolving fields and regions such as sub-Saharan Africa. In these contexts, publication lags and the under-reporting of null or context-specific findings could otherwise distort the evidence base. Including preprints also mitigates the risk of publication bias. Consistent with scoping review methodology, we did not exclude studies based on quality appraisal (37). We retained one preprint, a secondary analysis of the mHealth-HPVac study (41), which met our eligibility criteria. Studies were excluded if the intervention was not delivered via mobile technology, if the focus was on malignancies other than cervical cancer, if the source was a commentary or opinion piece lacking empirical or programmatic data, or if the study was conducted outside Africa. The study selection process was reported using a PRISMA-ScR flow diagram (Figure 1).

**Figure 1:**
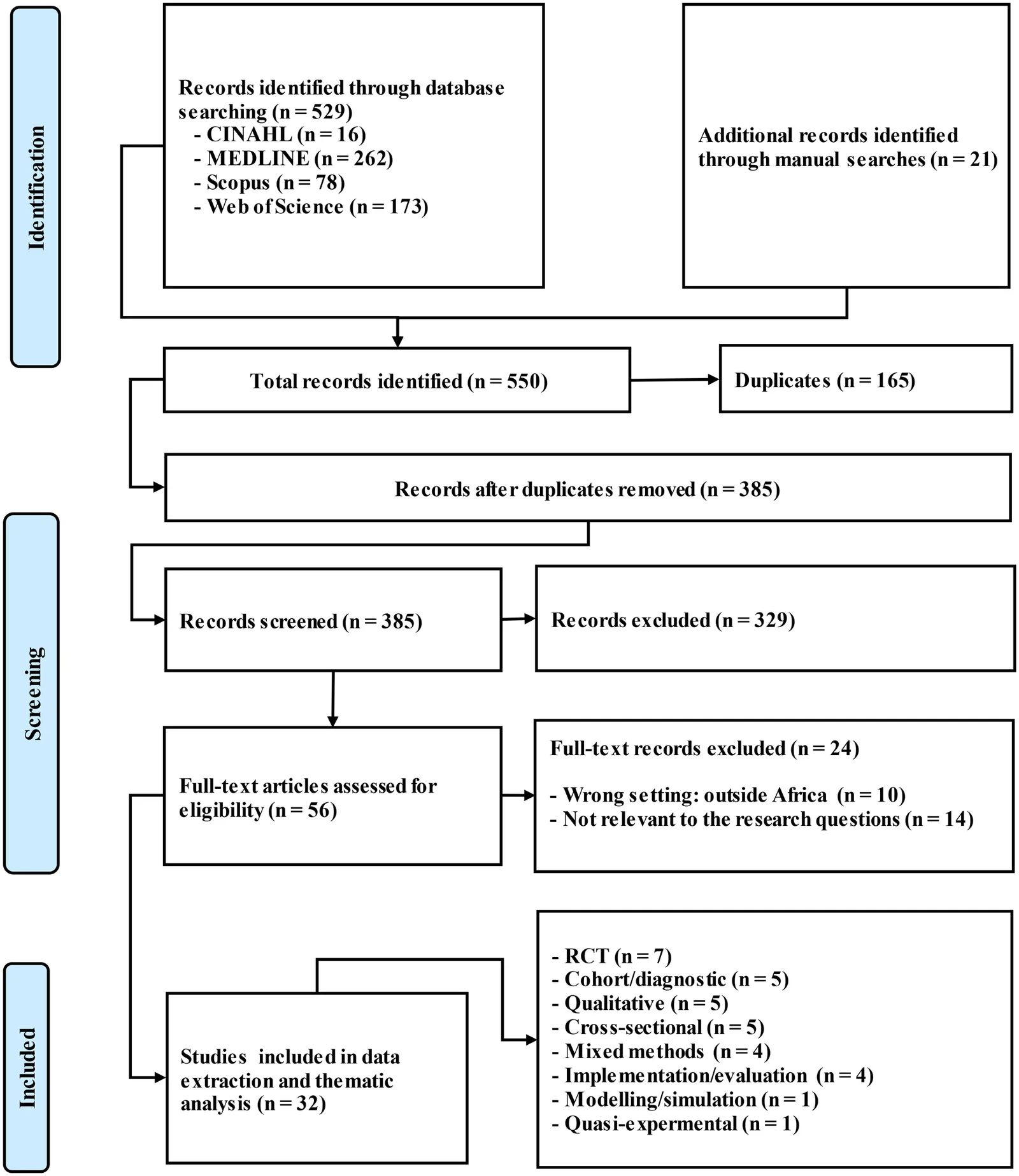
PRISMA-ScR flow diagram.

The search process retrieved 550 records, with 529 from database searches and 21 from manual searches. The database search identified records from various sources, including CINAHL (*n* = 16), MEDLINE (*n* = 262), Scopus (*n* = 78), and Web of Science (*n* = 173). After eliminating 165 duplicate records, 385 unique records remained for further examination. Subsequent screening of titles and abstracts excluded 329 records, leaving 56 full-text articles for eligibility assessment. Of these, 24 articles were excluded because they were conducted outside Africa (*n* = 10) or were not relevant to the research questions (*n* = 14). Ultimately, 32 studies were included in the final review and underwent data extraction and thematic analysis. The studies encompassed a variety of research designs, including randomized controlled trials (*n* = 7), cohort/diagnostic studies (*n* = 5), qualitative studies (*n* = 5), mixed-methods studies (*n* = 4), implementation or evaluation studies (*n* = 4), cross-sectional studies (*n* = 5), one modeling/simulation study, and one quasi-experimental study.

### 2.4 Stage 4: charting the data

A standardized data-charting instrument was developed collaboratively by the review team and piloted on a subset of 5 included studies. It was then iteratively refined as proposed by Levac et al. (37). Extracted variables comprised author and year of the study, country, study title, population and sample size, design, the stage of the cervical cancer care continuum addressed, the devices and platforms used, type of mHealth intervention, outcomes measured, and barriers and facilitators. The charting instrument was piloted independently by two reviewers to ensure consistency prior to full-scale data extraction. Table 1 provides an overview of the characteristics of each included study, comprising demographic characteristics, mHealth tools and platforms used, and the cervical cancer care continuum addressed. As individual studies could address multiple stages of the care continuum, outcome domains, facilitators, or challenges, the categories were not mutually exclusive; consequently, the total category counts may exceed the 32 included studies.

**Table 1:** Characteristics of included studies on mHealth interventions in cervical cancer prevention and control

Author, year	Country	Study title	Study population	Sample Size (n)	Study design	mHealth tools/platform	Care continuum stage
Addo-Lartey et al. (17), 2024	Ghana	“Effectiveness of a culturally tailored text messaging program for promoting cervical cancer screening in Accra, Ghana: a quasi-experimental trial”	Women aged 18–39 who had never had a Pap smear, recruited from banks and nearby communities, Greater Accra.	*n* = 88	Quasi-experimental	Mobile phones receiving SMS messages	Screening
Asare et al. (18), 2025	Ghana	“Culturally adapting and evaluating an evidence-based communication intervention with HPV self-sampling to improve cervical cancer screening among women living with HIV in Ghana: a mixed-methods Study”	Women living with HIV (WLHIV), health care providers (HCPs), and community members participating in a Nominal Group Technique adaptation process and subsequent evaluation.	*n* = 36 (stakeholder advisory board [14 WLHIV, 11 HCPs, 11 community members]) *n* = 45 (evaluation [30 WLHIV and 15 HCPs])	Mixed methods	Mobile phone; WhatsApp audio messages in English and Fante/Akan.	Screening
Asgary et al. (20), 2016	Ghana	“mHealth to train community health nurses in Visual Inspection with Acetic Acid for cervical cancer screening in Ghana”	Community health nurses (CHNs) trained in smartphone-supported VIA, and women screened at community health centers.	*n* = 169 women *n* = 15 CHNs	Cohort study/diagnostic-accuracy	Smartphones; SMS delivered via WhatsApp for CHN training and image transfer	Screening
Asgary et al. (21), 2019	Ghana	“Acceptability and implementation challenges of smartphone-based training of community health nurses for visual inspection with acetic acid in Ghana: mHealth and cervical cancer screening”	CHNs performing smartphone-based VIA/cervicography, women undergoing VIA/cryotherapy, and their supervisors (nurse supervisor and expert reviewer).	*n* = 38 (15 CHNs+21 women+2 supervisors)	Qualitative	Smartphones; image-based training and case-review platform	Screening
Asgary et al. (22), 2020	Eswatini	“Evaluating smartphone strategies for reliability, reproducibility, and quality of VIA for cervical cancer screening in the Shiselweni region of Eswatini: a cohort study”	Women attending VIA screening in the Shiselweni region, 2016–2018 (49% HIV-positive).	*n* = 4,247	Cohort study/diagnostic	Smartphones; cervical images transmitted to a remote expert reviewer	Screening
Asirwa et al. (42), 2025	Kenya	“A prospective model of the potential clinical and economic impact of cervical cancer screening supported by a mobile phone app”	Simulated cohort of women undergoing HPV-based cervical cancer screening in Kenya (modeled comparison of app-supported vs conventional care).	*n* = 20,000	Modeling/simulation	Smartphone-based mobile app (modeled/simulated study)	Diagnosis & Treatment
Chapola et al. (55), 2025	Malawi	“Exploring perceived barriers and facilitators to using mobile phone technology for delivering HPV test results in Malawi: a qualitative study with HPV-Screened women, healthcare providers, and health officials”	HPV-screened women, healthcare providers, and district health office officials from multiple facilities, Malawi.	*n* = 35 (20 women, 13 HCWs, 2 officials)	Qualitative	Mobile phones; voice calls and SMS for delivery of HPV screening results.	Diagnosis & Treatment
Choi et al. (43), 2024	Kenya	“The impact of text message reminders on cryotherapy uptake among women testing positive for HPV in western Kenya: a prospective cohort study”	Women aged 25–65 attending community health campaigns for HPV-based screening, western Kenya.	*n* = 3,303	Cohort study/diagnostic	Mobile phones; SMS and phone calls (standard vs enhanced text message arms).	Diagnosis & Treatment
DeGregorio et al. (52), 2016	Cameroon	“Prevalence, predictors, and same day treatment of positive VIA enhanced by digital cervicography and histopathology results in a cervical cancer prevention program in Cameroon”	Women attending the CBCHS Women's Health Program cervical cancer prevention services, Cameroon (2007–2014).	*n* = 44,979	Implementation/program evaluation	Dedicated (non-smartphone) digital camera; VIA enhanced by digital cervicography (VIA-DC).	Screening & Treatment
Domgue et al. (25), 2022	Cameroon	“Development, implementation, and evaluation of a distance learning and telementoring program for cervical cancer prevention in Cameroon”	Health-care practitioners and support staff in Cameroon, including prior and new ECHO telementoring participants.	*n* = 75	Cross-sectional	Computers and/or smartphones for videoconferencing; Zoom software (Zoom Video Communications) used for monthly Project ECHO tele-mentoring sessions	Workforce/system capacity
Holmström et al. (44), 2021	Kenya	“Point-of-care digital cytology with artificial intelligence for cervical cancer screening in a resource-limited setting”	HIV-positive women aged 18–64 attending a single rural clinic, Kenya; cervical (Papanicolaou) smears collected Sept 2018–Sept 2019.	*n* = 740	Cohort study/diagnostic	Portable slide scanner; images uploaded via mobile network to a cloud server; deep-learning system (DLS) accessed via computer/cloud interface.	Screening
Huchko et al. (45), 2019	Kenya	“How providing cervical cancer screening results via cell phone affects patient follow-up rates in western Kenya”	Women who underwent HPV-based cervical cancer screening in rural western Kenya, notified of results via different modalities.	*n* = 4,947	Mixed methods	Mobile phones; text message and phone call arms	Screening & Treatment
Kabukye et al. (32), 2023	Uganda	“Implementing smartphone-based telemedicine for cervical cancer screening in Uganda: qualitative study of stakeholders’ perceptions”	Health workers at screening clinics and women undergoing cervical cancer screening, Uganda.	*n* = 42 (27 HCWs+15 women)	Qualitative	Smartphones; custom-built smartphone-based telemedicine system for cervical image capture, data sharing, and remote case review	Screening
Kitaka et al. (27), 2025	Uganda	“SEARCH Study: text messages and automated phone reminders for HPV vaccination in Uganda: randomized controlled trial”	Caregivers of adolescents needing a first or second HPV vaccine dose, recruited from clinics in Kampala, Uganda.	*n* = 154	RCT	Mobile phones; SMS or automated voice call (caregiver's choice) for HPV vaccination reminders	Primary Prevention
Lee et al. (56), 2024	Malawi	“Mobile health–delivered narrative intervention to increase cervical cancer screening among Malawian women living with HIV: A pilot randomized controlled trial”	Malawian women living with HIV, randomized to three education-delivery arms (narrative video, non-narrative video, in-person materials).	*n* = 179	RCT	Tablet computers; pre-recorded video delivery (narrative and non-narrative arms).	Screening
Linde et al. (48), 2020	Tanzania	“Effectiveness of one-way text messaging on attendance to follow-up cervical cancer screening among human papillomavirus–positive Tanzanian women (Connected2Care): parallel-group randomized controlled trial”	HPV-positive women across 3 hospitals in Tanzania, part of the Connected2Care trial.	*n* = 705 enrolled (685 analyzed)	RCT	Mobile phones; one-way SMS reminders (Connected2Care trial).	Screening
Løkke et al. (49), 2022	Tanzania	“Acceptability of text messages and knowledge change for cervical cancer screening: a Tanzanian mixed methods study”	HPV-positive women in the intervention arm of the Connected2Care trial in Tanzania.	*n* = 115	Mixed methods	Mobile phones; one-way SMS reminders (Connected2Care trial).	Screening
Moodley et al. (57), 2019	South Africa	“Exploring the feasibility of using mobile phones to improve the management of clients with cervical cancer precursor lesions”	Women eligible for Pap smear at public-sector services, Cape Town, South Africa (18–30y from HIV clinics; ≥30y from PHC clinics), plus providers/managers and colposcopy clients.	*n* = 371 surveyed (+10 IDIs+27 FGD) [Results are based on 364]	Mixed methods	Mobile phones (basic/feature phones and smartphones); SMS identified as the primary intended function for result/reminder delivery.	Diagnosis & Treatment
Mungo et al. (31), 2021	Kenya	“Feasibility and acceptability of smartphone-based cervical cancer screening among HIV-positive women in western Kenya”	Women living with HIV aged 25–49, HPV-positive, attending an HIV clinic, Kenya.	*n* = 164	Cohort study/diagnostic	Smartphone camera used to capture 3+ cervical images per participant	Screening
Mwandacha et al. (54), 2024	Uganda	“Investigating the healthcare-seeking behaviors of mobile phone users to improve cervical cancer screening in rural Uganda”	Women aged 30–49 living in the South Busoga Forest reserve with no screening/treatment in the previous 5 years, Uganda.	n = 1,434	Cross-sectional	Mobile phones (formative access/ownership study).	Screening
Nkfusai et al. (53), 2025	Cameroon	“Impact of telephone reminders on posttreatment follow-up among women treated for cervical precancers in Cameroon: a randomized controlled trial”	Women treated for VIA/VILI-positive cervical precancer who missed scheduled post-treatment follow-up within 3 years, Cameroon.	*n* = 203 enrolled (185 analyzed)	RCT	Mobile phones; telephone calls and SMS reminders for post-treatment follow-up.	Post-treatment Follow-up
Ogunsola et al. (30), 2023	Nigeria	“AVIVA: a telehealth tool to improve cervical cancer screening in resource-constrained settings”	Women living with HIV receiving VIA-based cervical screening across APIN-supported facilities in 7 states, Nigeria.	*n* = 29,262	Implementation/program evaluation	Smartphones with camera, provided to case-finders; AVIVA app (APIN VIA Visual Application) used for image capture and transmission to remote gynecologist reviewers; SMS notifications for reviewers and case finders	Screening & Treatment
Okunade et al. (19), 2021	Nigeria	“Impact of mobile technologies on cervical cancer screening practices in Lagos, Nigeria (mHealth-Cervix): A randomized controlled trial”	Sexually active women aged 25–65, not strictly adherent to Pap screening recommendations, owning a personal mobile phone, Lagos, Nigeria.	*n* = 200	RCT	Mobile phones; SMS reminders	Screening
Okunade et al. (28), 2025	Nigeria	“A randomized controlled trial of impact of mHealth technologies on human papillomavirus vaccination uptake in mothers of vaccine-eligible girls in Lagos, Nigeria (mHealth-HPVac)”	Mothers of unvaccinated girls aged 9–14 attending GOP clinics at Lagos University Teaching Hospital, Nigeria.	*n* = 180	RCT	Mobile phones; MultiTexter Bulk SMS platform, an automated message-delivery system with delivery-confirmation analytics	Primary Prevention
Okunade et al. (41), 2026	Nigeria	“Effect of socioeconomic status on cervical cancer screening behavior among mothers of adolescent girls in Lagos, Nigeria: A secondary analysis of the mHealth-HPVac study”	Mothers of unvaccinated girls aged 9–14 with complete sociodemographic/screening data (secondary analysis of mHealth-HPVac baseline data), Lagos, Nigeria.	*n* = 180	Cross-sectional	Mobile phone (secondary analysis of baseline ownership data; no active intervention).	Screening
Onyango et al. (29), 2025	Kenya	“Text messaging to improve uptake of human papillomavirus vaccination: a study among adolescent girls living with HIV in Kisumu County”	Adolescent girls living with HIV aged 10–14, HPV vaccine-naïve, attending comprehensive care clinics, Kisumu County, Kenya.	*n* = 152 enrolled (151 analyzed)	RCT	Mobile phones (guardians’); weekly SMS reminders.	Primary Prevention
Sibomana et al. (24), 2025	Rwanda	“Health worker experiences on the integration of digital health tools for HPV vaccination and cervical cancer services in Rwanda”	Key informant health workers (nurses, doctors, data managers, program/M&E officers), Rwanda.	*n* = 15	Qualitative	Mobile/computer devices running the e-tracker (HPV vaccination) and mUzima (screening/treatment) applications, integrated with the national OpenMRS electronic health record platform.	Workforce/system capacity
Stocks et al. (23), 2022	Kenya	“Iterative development of a mobile phone app to support community health volunteers during cervical cancer screening in western Kenya: Qualitative study”	Community health volunteers (CHVs) and clinicians from urban and rural western Kenya engaged in HPV-based screening.	*n* = 18	Qualitative	Smartphones (Android); “mSaada” app developed by a Duke University computer science student team and iteratively refined with community health volunteer and clinician feedback.	Screening
Stocks et al. (46), 2022	Kenya	“Mobile phone ownership and use among women screening for cervical cancer in a community-based setting in western Kenya: Observational study”	Women aged 25–65 attending community health campaigns for HPV self-collection, western Kenya.	*n* = 3,299	Cross-sectional	Mobile phone, SMS, phone call, and home-visit notification compared	Screening & Treatment
Tonui et al. (47), 2025	Kenya	“Digital cervicography using mobile phones with real-time consultation (DCRC) to improve performance of Visual Inspection with Acetic Acid (VIA) in cervical cancer screening of HIV-infected women: A cross-sectional study”	HIV-positive women at two rural outreach clinics (Webuye and Chulaimbo), Kenya.	*n* = 200	Cross-sectional	Smartphones with camera; cervical images shared with gynecologic oncologists for real-time telemedicine consultation	Screening
Yeates et al. (51), 2016	Tanzania	“Evaluation of a smartphone-based training strategy among health care workers screening for cervical cancer in northern Tanzania: The Kilimanjaro method”	Non-physician providers (nurses, assistant medical officers) trained in VIA, and women they screened, northern Tanzania.	*n* = 1,072	Implementation/program evaluation	Smartphones; a free, commercially available smartphone application used for real-time cervical image transfer across a closed user group of regional experts (“The Kilimanjaro Method”)	Screening
Yeates et al. (50), 2020	Tanzania	“Smartphone-enhanced training, QA, monitoring, and evaluation of a platform for secondary prevention of cervical cancer: opportunities and challenges to implementation in Tanzania”	National-scale cervical cancer screening program (CECAP) population across 5 regions of Tanzania.	*n* = 10,545	Implementation/program evaluation	Smartphones; SEVIA (smartphone-enhanced VIA) platform for real-time, secure image sharing, remote supportive supervision, data monitoring, and evaluation, integrated into the Tanzania National Cervical Cancer Prevention Program (CECAP).	Screening & Treatment

### 2.5 Stage 5: collating, summarizing, and reporting results

Consistent with Stage 5 of the methodological framework by Levac et al. (37), a two-step approach was employed. First, a descriptive numerical summary was generated. This involved tabulating the frequency of included studies based on country, intervention type, study design, publication year, and the stage of the cervical cancer care continuum addressed, as extracted using the standardized charting instrument (Table 1). Second, a narrative synthesis of the same charted data was undertaken to characterize the nature and extent of the evidence in greater depth. AH and GM familiarized themselves with the charted data, and then they organized and summarized findings using categories derived directly from the scoping review's *a priori* research sub-questions: intervention type, care continuum stage, outcome domain, facilitator, and challenge. These groupings were integrated into the narrative synthesis presented in the results section. The process involved collaborative work followed by joint resolution of discrepancies through iterative discussion. This approach aligns with the guidance by Levac et al. (37) that Stage 5 should combine a descriptive numerical summary with a qualitative “descriptive analytical” method appropriate to the review's aims.

## 3 Results

### 3.1 Study demographic characteristics

This scoping review identified 32 eligible studies concerning mHealth interventions for cervical cancer screening, diagnosis, and treatment in Africa. Geographically, studies were predominantly concentrated in East and West Africa. Kenya contributed the largest proportion of studies (*n* = 9) (23, 29, 31, 42–47), followed by Ghana (17, 18, 20, 21), Tanzania (48–51), and Nigeria (21, 28, 30, 41) (*n* = 4 each), Cameroon (25, 52, 53) and Uganda (27, 32, 54) (*n* = 3 each), and Malawi (*n* = 2) (55, 56). Single studies originated from Eswatini (22), South Africa (57), and Rwanda (24) (Table 2).

**Table 2:** Summary of study characteristics

Variables	Category	Number of studies (n)	Studies
Countries	Kenya	*n* = 9	(23, 29, 31, 42–47)
	Ghana	*n* = 4	(17, 18, 20, 21)
	Tanzania	*n* = 4	(48–51)
	Nigeria	*n* = 4	(19, 28, 30, 41)
	Cameroon	*n* = 3	(25, 52, 53)
	Uganda	*n* = 3	(27, 32, 54)
	Malawi	*n* = 2	(55, 56)
	Eswatini	*n* = 1	(22)
	South Africa	*n* = 1	(57)
	Rwanda	*n* = 1	(24)
Design	RCT	*n* = 7	(19, 27–29, 48, 53, 56)
	Cohort study/diagnostic	*n* = 5	(20, 22, 31, 43, 44)
	Qualitative	*n* = 5	(21, 23, 24, 32, 55)
	Mixed methods	*n* = 4	(18, 45, 49, 57)
	Implementation/program evaluation	*n* = 4	(30, 50–52)
	Cross-sectional	*n* = 5	(25, 41, 46, 47, 54)
	Modeling/simulation	*n* = 1	(42)
	Quasi-experimental	*n* = 1	(17)
Study population type	General screening-eligible women	*n* = 12	(17, 19–22, 32, 45, 46, 51, 52, 54, 57)
	Health care workers/providers	*n* = 10	(18, 20, 21, 23–25, 32, 51, 55, 57)
	Women living with HIV (WLHIV)	*n* = 6	(18, 30, 31, 44, 47, 56)
	HPV-positive women	*n* = 4	(43, 48, 49, 55)
	Caregivers of HPV-vaccine-eligible adolescents	*n* = 3	(27, 28, 41)
	Simulated/modeled population	*n* = 1	(42)
	Women previously treated for cervical precancer	*n* = 1	(53)
	Adolescent girls living with HIV (vaccine-eligible)	*n* = 1	(29)
	National/program-level population	*n* = 1	(50)
Sample size band	<50	*n* = 5	(21, 23, 24, 32, 55)
	50–199	*n* = 11	(17, 18, 20, 25, 27–29, 31, 41, 49, 56)
	200–999	*n* = 6	(19, 44, 47, 48, 53, 57)
	1,000–4,999	*n* = 6	(22, 43, 45, 46, 51, 54)
	≥5,000	*n* = 4	(30, 42, 50, 52)

General screening-eligible women were the most frequently studied population (*n* = 12) (17, 19–22, 32, 45, 46, 51, 52, 54, 57). Healthcare workers and providers, including community health nurses, community health volunteers, and clinicians, formed the second largest group (*n* = 10) (18, 20, 21, 23–25, 32, 51, 55, 57), reflecting the use of mHealth for training, quality assurance, and tele-mentoring alongside client-facing interventions. Populations at elevated risk of cervical cancer were also well represented. Six studies focused on women living with HIV (WLHIV) (18, 30, 31, 44, 47, 56), and four on HPV-positive women, largely in the context of result delivery, follow-up, and treatment uptake (43, 48, 49, 55). A smaller body of work addressed primary prevention through HPV vaccination, targeting caregivers of vaccine-eligible adolescents (*n* = 3) (27, 28, 41) and, in one study, adolescent girls living with HIV (29). Single studies also examined women previously treated for cervical precancer (53), a national or program-level population (50), and a simulated (modeled) population used to project the clinical and economic impact of app-supported screening (42).

Sample sizes varied considerably, ranging from 15 (24) to 44,979 participants (52). Five studies enrolled fewer than 50 participants (21, 23, 24, 32, 55), eleven enrolled 50–199 (17, 18, 20, 25, 27–29, 31, 41, 49, 56), six enrolled 200–999 (19, 44, 47, 48, 53, 57), six enrolled 1,000–4,999 (22, 43, 45, 46, 51, 54), and four exceeded 5,000 (30, 42, 50, 52). Methodologically, randomized controlled trials were the most frequent design (*n* = 7) (19, 27–29, 48, 53, 56), followed by five cohort/diagnostic studies (20, 22, 31, 43, 44), five qualitative studies (21, 23, 24, 32, 55), four mixed-methods studies (18, 45, 49, 57), four implementation/program-evaluation studies (30, 50–52), five cross-sectional designs (25, 41, 46, 47, 54), a single modeling/simulation design (42), and a single quasi-experimental design (17).

### 3.2 Care continuum stages targeted by mHealth interventions in cervical cancer prevention and control

mHealth interventions for cervical cancer in Africa vary considerably (Table 3). Screening interventions were most common (*n* = 22), appearing in nearly all included countries (17–23, 30–32, 41, 44–52, 54, 56), followed by diagnosis and treatment (*n* = 9) (30, 42, 43, 45, 46, 50, 52, 55, 57). Same-day treatment completion rates varied from 31.1% in Cameroon (52) to 48.9% in Tanzania (50). In South Africa, this stage was expanded to include qualitative focus groups with colposcopy clinic attendees and interviews with providers and managers, addressing both clinical procedures and patient experience (57). Primary prevention through HPV vaccination reminders was reported in three studies (27–29), while post-treatment follow-up was the least represented stage (53). Workforce and system-capacity interventions were reported in two studies, in Cameroon and Rwanda (24, 25). Stage categories were not mutually exclusive; for instance, five studies in Kenya (45, 46), Nigeria (30), Tanzania (50), and Cameroon (52) addressed screening, diagnosis, and treatment, so the stage totals exceed the 32 included studies.

**Table 3:** Care continuum stages targeted by mHealth interventions in cervical cancer prevention and control

Care continuum stage	Key findings	Number of studies (n)	Studies
Screening	- Point of cervical cancer screening—VIA, HPV-based testing, and digital cervicography.	*n* = 22	(17–23, 30–32, 41, 44–52, 54, 56)
	- Detecting precancerous or cancerous lesions in women presenting for screening.		
	- Smartphone-based image capture with tele-expert review to support diagnostic quality.		
	- SMS or app-based reminders and education to improve screening attendance.		
Diagnosis & Treatment	- Same-day, screen-and-treat programs.	*n* = 9	(30, 42, 43, 45, 46, 50, 52, 55, 57)
	- Enhanced versus standard SMS message for cryotherapy uptake.		
	- SMS versus phone call versus home-visit notification for linking HPV-positive women to treatment.		
	- A modeled app-based results-communication strategy.		
	- App-based system for communicating results and coordinating treatment follow-up, estimating cost-effectiveness.		
Primary Prevention	- HPV vaccination of adolescent girls as the primary preventive intervention against cervical cancer	*n* = 3	(27–29)
	- RCTs testing mobile-phone-delivered reminders against usual care, allowing direct comparison of vaccination uptake between arms.		
	- Reminding caregivers of a first or second HPV vaccine dose via SMS or automated voice call (participant's choice)		
	- Reminding mothers of unvaccinated girls aged 9–14 attending clinics.		
	- Delivering weekly SMS messages to guardians over a six-month period covering HPV, vaccination, and cervical cancer information.		
Workforce and system capacity	- Live videoconferencing tele-mentoring program (a Project ECHO model) connecting health-care practitioners and support staff with specialist faculty for ongoing case-based education in cervical cancer prevention and treatment.	*n* = 2	(24, 25)
	- Digital registries (e-tracker, mUzima) for cervical cancer screening and treatment data, both integrated with the national OpenMRS platform.		
	- System-level data quality, tracking, and integration.		
Post-treatment Follow-up	- Adherence to scheduled follow-up visits after treatment for cervical precancer.	*n* = 1	(53)
	- Telephone-plus-SMS reminders to ensure women return for surveillance of treatment success or recurrence.		

### 3.3 Types of mHealth interventions in cervical cancer prevention and control

This scoping review identified a heterogeneous array of mHealth interventions for cervical cancer prevention and control, categorized into five distinct types based on their functional mechanisms, geographic distribution, and target populations (Table 4).

**Table 4:** Types of mHealth interventions in cervical cancer prevention and control

mHealth intervention	Targeted population	Description of mHealth intervention	Countries (n)	Number of studies (n)	Studies
Messaging, Voice-Based Communication & Awareness-Raising	- General screening-eligible women - HPV-positive women awaiting treatment/follow-up - Caregivers of HPV-vaccine-eligible adolescents - Adolescent girls living with HIV - Women previously treated for cervical precancer - Women living with HIV - Health care workers, jointly with patients	- One-way or two-way SMS text messages, audio messages, or voice calls sent directly to women’s or caregivers’ mobile phones. - Used for health education, appointment/screening reminders, or delivery of screening/HPV results. - Includes studies that directly compared two or more communication channels (e.g., SMS vs phone call vs home visit) - Patient-directed awareness-raising: pre-recorded narrative or non-narrative videos delivered on tablets as an alternative to in-person health talks	- Kenya (*n* = 4) - Ghana (*n* = 2) - Tanzania (*n* = 2) - Nigeria (*n* = 2) - Malawi (*n* = 2) - Uganda (*n* = 1) - Cameroon (*n* = 1)	*n* = 14	(17–19, 27–29, 43, 45, 46, 48, 49, 53, 55, 56)
Image-Based Diagnostic & Tele-Expert Review	- Health care workers/providers, jointly with the general screening-eligible women (dual-population studies) - General screening-eligible women studied alone, with no paired provider population - Women living with HIV undergoing screening - National program-level population	- A frontline provider captures a cervical image (VIA/VIA-DC/cervicography) or cytology slide using a smartphone camera or digital scanner. - The image is transmitted in real time or asynchronously to an off-site reviewer — either a human expert (gynecologist, oncologist, pathologist) or an artificial-intelligence/deep-learning model — for diagnostic confirmation, mentorship, or automated interpretation.	- Kenya (*n* = 3) - Ghana (*n* = 2) - Tanzania (*n* = 2) - Eswatini (*n* = 1) - Cameroon (*n* = 1) - Uganda (*n* = 1) - Nigeria (*n* = 1)	n = 11	(20–22, 30–32, 44, 47, 50–52)
Digital Applications for Decision-Support & Care Coordination	- Health care workers/providers (community health volunteers, clinicians) - Simulated/modeled population	- A dedicated mobile or web application supporting clinical decision-making, results communication, or care coordination. - Structured electronic data capture, protocol guidance, or navigation support, distinct from simple messaging or image-transfer tools.	- Kenya (*n* = 2) - Rwanda (*n* = 1)	n = 3	(23, 24, 42)
Provider Education & Training	- Health care providers	- A brief classroom course (two weeks in Ghana, six days in Tanzania) followed by continuing case-based expert feedback, given through smartphone image-sharing (Ghana, Tanzania); or monthly ECHO video-conference tele-mentoring (Cameroon).	- Cameroon (*n* = 1) - Tanzania (*n* = 2) - Ghana (*n* = 2)	n = 5	(20, 21, 25, 50, 51)
Baseline and feasibility studies	- General screening-eligible women - Caregivers of HPV-vaccine-eligible adolescents - Health care workers	- Assessment of mobile phone ownership, access, or interest in future mHealth use as a baseline/feasibility exercise, without deploying an actual mHealth intervention to participants.	- South Africa (*n* = 1) - Uganda (*n* = 1) - Nigeria (*n* = 1)	n = 3	(41, 54, 57)

#### 3.3.1 Messaging, voice-based communication and awareness-raising

Messaging, voice-based communication, and awareness-raising constituted the most prevalent intervention category (*n* = 14), with studies conducted across seven countries — Kenya, Ghana, Tanzania, Nigeria, Malawi, Uganda, and Cameroon (17–19, 27–29, 43, 45, 46, 48, 49, 53, 55, 56). This category engaged a broad spectrum of populations, including women eligible for general screening (17, 19, 45, 46), HPV-positive women awaiting treatment or follow-up (43, 48, 49), caregivers of HPV-vaccine-eligible adolescents (27, 28), adolescent girls living with HIV (29), and women previously treated for cervical precancer (53). A smaller subset of studies also examined healthcare workers alongside their patients (18, 55). Interventions comprised one-way or two-way SMS text messages (17, 19, 28, 29, 43, 48, 49), audio messages delivered via WhatsApp (18), or voice calls, either alone or combined with SMS (27, 53, 55), all delivered directly to mobile phones. These interventions were used for health education and correction of locally documented misconceptions (17, 18), appointment or screening reminders (19), HPV vaccination reminders (27–29), post-treatment follow-up reminders (53), or the delivery of screening and HPV results (43, 45, 46, 55). Notably, this category also encompassed studies that directly compared multiple communication channels, including SMS, phone calls, home visits, and clinic notifications (45, 46). In addition, this category included patient-directed awareness-raising: in Malawi, pre-recorded narrative and non-narrative videos were delivered to women living with HIV via tablet as an alternative to in-person health talks (56).

#### 3.3.2 Image-based diagnostic and tele-expert review

Image-based diagnostic and tele-expert review represented a similarly substantial category (*n* = 11) with comparable geographic breadth, spanning seven countries: Kenya, Ghana, Tanzania, Eswatini, Cameroon, Uganda, and Nigeria (20–22, 30–32, 44, 47, 50–52). Its population profile was more clinically oriented: four studies targeted healthcare workers and providers jointly with the general screening-eligible women they served (20, 21, 32, 51), while two further studies recruited only general screening-eligible women, without a paired provider population (22, 52). This category also included a notable subset of studies recruiting women living with HIV specifically for diagnostic evaluation (30, 31, 44, 47), as well as one study reporting outcomes at the level of an entire national screening program rather than a defined enrolled cohort (50). The underlying mechanism involved a frontline provider capturing a cervical image with visual inspection with acetic acid enhanced by digital cervicography (VIA-DC) (52) or by real-time-consultation cervicography (47), or a cytology slide, using a smartphone camera (20–22, 30–32, 47, 50, 51), a dedicated non-smartphone digital camera (52), or a portable slide scanner (44). This image was then transmitted to an off-site reviewer—either a human expert such as a gynecologist or oncologist (30, 47) or a cytopathologist serving as the reference standard (44), or an artificial intelligence/deep-learning model (44)—for diagnostic confirmation and mentorship (20–22, 51) or automated interpretation (44).

#### 3.3.3 Digital applications for decision support and care coordination

Digital applications for decision support and care coordination (*n* = 3) were geographically restricted to Kenya and Rwanda (23, 24, 42) and targeted almost exclusively healthcare workers and providers, including community health volunteers and clinicians (23, 24), alongside one study employing a wholly simulated population without human subjects (42). This category comprised dedicated mobile or web-based applications supporting clinical decision-making and protocol guidance for HPV-based screening (23), results communication and treatment-linkage coordination modeled at population scale (42), or national-level care coordination through vaccination- and screening-tracking registries integrated with an existing electronic health record platform (24). These applications facilitated structured electronic data capture (23, 24), protocol guidance, or navigation support (23), making them functionally distinct from simple messaging or image-transfer tools.

#### 3.3.4 Provider education and training

Provider education and training was reported in five studies (20, 21, 25, 50, 51). These types of interventions followed a blended, mentorship-based model: a brief conventional course was paired with ongoing case-based feedback from experts, delivered through smartphone image-sharing (20, 21, 50, 51) or through monthly video-conference tele-mentoring using Project ECHO (25). In Ghana, 15 community health nurses completed a two-week course, followed by three months of smartphone image-based mentorship (20). They screened 169 women, achieving 95% overall agreement with an expert reviewer. The mean agreement per nurse was 89.6%, with a kappa of 0.67, though agreement on positive cases was only 61.5%, attributed to the low number of positive cases. Qualitative findings from Asgary et al. (21) indicated that nurses valued mentorship, exposure to positive cases, and peer support. They reported increased confidence and recommended smaller training groups.

In Tanzania, five providers who completed a six-day course screened 1,072 women. Disagreement with experts decreased from approximately 10% to under 3% within a month, resulting in 96.8% overall agreement (51). When scaled up across five regions in Tanzania, 51 providers at 24 facilities screened 10,545 women; concordance remained around 90%, over 50 providers graduated, and VIA positivity increased from 4.0% to 6.2% (50). However, concordance fluctuated with the integration of new providers and reviewers, and some feedback took 24–72 h (50). In Cameroon, monthly ECHO tele-mentoring was associated with significantly higher satisfactory knowledge (53.8% vs. 4.5%) and greater use of VIA, cryotherapy, and thermal ablation among attendees compared to newcomers (25), although over half of participants reported connectivity issues.

#### 3.3.5 Baseline and feasibility studies (descriptive)

Baseline and feasibility studies (*n* = 3) were conducted across three countries, namely South Africa, Uganda, and Nigeria (41, 54, 57), and assessed mobile phone ownership (54, 57), access in a remote, underserved setting (54), or interest in prospective SMS-based screening reminders and result delivery (57). These were conducted as baseline or feasibility exercises, including a secondary analysis of trial baseline data (41), without deploying an active mHealth intervention. This category targeted general screening-eligible women (54, 57) and caregivers of HPV-vaccine-eligible adolescents (41), and, in one mixed-methods study, healthcare workers and managers alongside the surveyed women (57).

### 3.4 Devices and platforms used for mHealth intervention in cervical cancer prevention and control

In this review, mHealth tools and platforms included basic mobile phones, smartphones, digital cameras, tablets, computer-based videoconferencing, or cloud-based platforms (Table 5). Basic mobile phones, offering SMS and voice calls, were the most reported device (*n* = 13) (17, 19, 27–29, 43, 45, 46, 48, 49, 53, 55, 57). These support low-cost interventions such as reminder systems and results delivery. For example, Kitaka et al. (27) used SMS/call vaccination reminders in Uganda, with 62% opting for SMS and 38% for calls. Nkfusai et al. (53) used phone-plus-SMS for follow-up in Cameroon. High comfort with SMS was reported in Kenya, where Choi et al. (43) found 92.5% of women receiving text reminders owned phones and were comfortable with SMS.

**Table 5:** Devices and platforms used for mHealth interventions in cervical cancer prevention and control

Device or platform	Description	Number of studies (n)	Studies
Basic/feature mobile phone	- Standard mobile phones capable of sending/receiving SMS text messages and voice calls.	*n* = 13	(17, 19, 27–29, 43, 45, 46, 48, 49, 53, 55, 57)
	- Does not require smartphone capabilities, a data plan, or app installation — the most widely accessible device category in low-resource settings.		
Smartphone	- Camera- and/or app-capable mobile phones used to capture cervical images.	*n* = 12	(18, 20–23, 30–32, 42, 47, 50, 51)
	- Run dedicated mHealth applications.		
	- App-based data entry.		
	- Send multimedia content (WhatsApp audio).		
	- Real-time image transfer and mentorship.		
Computer/videoconferencing/cloud platform	- Desktop/laptop computers or cloud-based server infrastructure used for videoconferencing, AI image analysis, or centralized data-registry systems.	*n* = 3	(24, 25, 44)
	- Zoom videoconferencing software used to deliver monthly Project ECHO tele-mentoring sessions.		
	- Portable slide scanner to digitize cervical Papanicolaou smears, then the resulting whole-slide images are uploaded to a cloud server over mobile networks, where a deep-learning system analyzes them for atypical cervical cells.		
	- Computer-based national registry systems.		
Any mobile phone (unspecified)	- General mobile phone access and ownership without specifying or requiring a particular device type.	*n* = 2	(41, 54)
	- No active intervention was deployed to a specific platform in these studies.		
Digital camera (non-smartphone)	- A dedicated, non-smartphone digital camera used to capture cervical images for VIA-enhanced digital cervicography (VIA-DC).	*n* = 1	(52)
	- The camera was used to capture and document cervical images during screening visits, supporting both same-day clinical decision-making and later record review.		
Tablet	- Tablet computers used primarily to deliver pre-recorded video content to patients in a clinic or community setting.	*n* = 1	(56)

Smartphones, essential for image capture, mobile apps, or multimedia, were prevalent in mHealth interventions (*n* = 12) (18, 20–23, 30–32, 42, 47, 50, 51). Smartphone cameras were used for remote cervical image review in Ghana and Eswatini (20–22), Kenya (31, 47), and Tanzania (50, 51). In Nigeria, Ogunsola et al. (30) and, in Uganda, Kabukye et al. (32) extended this to telemedicine and case-review apps. In Kenya, “mSaada”, an Android app, was developed for community health volunteers, who cited prior smartphone familiarity as a comfort factor (23).

In contrast, computer, videoconferencing, or cloud-based platforms were reported in three countries (*n* = 3) (24, 25, 44). In Cameroon, Domgue et al. (25) used Zoom for Project ECHO tele-mentoring. In Kenya, digitized cervical smears were used with a portable scanner and uploaded to a cloud server for AI analysis, achieving high sensitivity (44). In Rwanda, Sibomana et al. (24) examined national digital registries integrated with OpenMRS. This suggests that this category is associated with specialized research or health-system partnerships, not just phone ownership. A dedicated digital camera (*n* = 1) was used only in Cameroon, where DeGregorio et al. (52) utilized a clinical database of 44,979 women screened for cervical cancer from 2007 to 2014 and identified that 9% were VIA-DC positive. This represents an earlier approach before smartphone-based cervicography. Tablets (*n* = 1) were used exclusively in Malawi, where Lee et al. (56) delivered pre-recorded video education, the only instance of a device used purely for passive content delivery. Two studies (*n* = 2) in Nigeria and Uganda (41, 54) assessed generic mobile phones without specifying device types.

### 3.5 Outcomes of mHealth interventions in cervical cancer prevention and control

The included studies revealed considerable variation in the frequency and consistency of mHealth effectiveness across ten countries (Table 6). Diagnostic accuracy and concordance were reported in nine studies (20, 22, 30, 31, 44, 47, 50–52). In Ghana, the community health nurses’ VIA diagnoses agreed with the expert reviewer's in 95% of cases (20). In Kenya, AI-supported digital cytology achieved sensitivity of 95.7%–100% and specificity of 78.4–84.7% for detecting atypical cervical cells against pathologist assessment across two evaluation sets (44). Feasibility and acceptability of the mHealth tools were equally prevalent (*n* = 9) (18, 21, 23, 31, 32, 46, 49, 55, 57). For instance, in Uganda, Kabukye et al. (32) reported that 80% (16 of 20) of nurses rated the usability of their smartphone telemedicine system as good. Similarly, in Tanzania, Løkke et al. (49) observed an increase in acceptability (mean change 0.22, *p* = .05). Treatment uptake and linkage to treatment (*n* = 9) showed heterogeneity across countries (30, 42, 43, 45, 46, 50, 52, 55, 57). Same-day treatment completion varied from 31.1% in Cameroon (52) to 48.9% in Tanzania (50). In Kenya, Huchko et al. (45) found that home visits achieved 45% treatment uptake, compared to 38% for text messages, 38% for phone calls, and 23% for clinic notifications (*p* < .001). Conversely, Choi et al. (43) reported no significant difference in treatment uptake between the standard text group (48/90, 53.3%) and the enhanced text group (22/41, 53.7%) among HPV-positive women, suggesting that the notification modality, rather than message content, influenced outcomes within the same country.

**Table 6:** Reported outcomes of the mHealth interventions in cervical cancer prevention and control

Outcome domain	Key findings	Number of studies (n)	Studies
Diagnostic accuracy and concordance	- Smartphone-cervicography training produced consistently high agreement: 95% total agreement between community health nurses and expert reviewers (κ = 0.67, 95% CI 0.45–0.88), rising to κ up to 0.79 with sustained mentorship, and 96.8% trainee–expert agreement within one month of training.	*n* = 9	(20, 22, 30, 31, 44, 47, 50–52)
	- AI-supported digital cytology achieved the highest accuracy figures in the review: 95.7–100% sensitivity and 78.4–84.7% specificity for detecting atypical cervical cells against pathologist assessment across two evaluation sets.		
	- Notably low CIN2 + sensitivity in one study (21.4–35.7%).		
Feasibility/acceptability of the mHealth tool	- Consistently high acceptability: 87–100% agreement.	*n* = 9	(18, 21, 23, 31, 32, 46, 49, 55, 57)
	- 80% (16 of 20) of nurses rated their smartphone telemedicine system as having good usability, agreeing or strongly agreeing on 10 of 14 System Usability Scale items.		
	- High acceptability (mean 22.84), feasibility (mean 22.40), and adoption potential (mean 21.73)		
	- Improved acceptability from baseline to follow-up (mean change 0.22, 95% CI 0.00–0.44, p = .05).		
	- 99.4% of women were comfortable having a cervical image taken.		
Treatment uptake and linkage to treatment	- Same-day treatment completion ranged from 31.1% to 48.9%.	*n* = 9	(30, 42, 43, 45, 46, 50, 52, 55, 57)
	- Notification modality had a measurable and statistically significant effect on treatment uptake in one trial: Huchko et al. (45) found home visits achieved 45% treatment uptake versus 38% for text message, 38% for phone call, and only 23% for clinic-based notification (p < .001).		
	- Enhanced SMS message (with additional educational content) did not significantly improve cryotherapy uptake compared with a standard SMS message among HPV-positive women.		
Screening uptake and attendance	- Mixed findings were seen in screening uptake, with some significance reported (51.0% vs 35.7%, p = .031) in Nigeria	*n* = 6	(17, 19, 41, 48, 54, 56)
	- Video-based education arms increased 2-month VIA uptake significantly vs in-person materials alone (51.0%/50.0% vs 35.0%, p = .01) in Malawi		
	- Mobile phone access was significantly associated with higher attendance at community outreach screening (87.3% vs 72.6% among women without access) and at health-center-based screening (96.9% vs 93.5%).		
Provider competency and confidence	- Smartphone-based mentorship was associated with rising trainee–expert agreement, from about 90% to 96.8% within one month in Tanzania, and nurses in Ghana reported greater confidence and stronger diagnostic and case-management competence.	*n* = 5	(20, 21, 25, 50, 51)
	- Expert feedback was fast in the small Tanzanian study: 52.2% of cases within 5 minutes (48.4% in the 1–5 minute band) and 61.2% within 10 minutes. In the national scale-up, only 45.6% were reviewed within 10 minutes, and 21.7% waited 24–72 hours.		
	- Community health nurses described the 3-month mentorship as essential for developing diagnostic and management skills, and they valued peer-to-peer support and case discussion as an additional way of learning.		
	- Project ECHO attendance was associated with higher self-reported use of VIA/VILI (63.2% vs 33.3%, p = .03) and greater knowledge than among newcomers, though this cross-sectional finding cannot establish that ECHO caused the difference.		
Program and health-system implementation outcomes	- Concordance improved substantially across program phases (26%→42% VIA+, 80%→96% VIA−).	*n* = 3	(24, 30, 50)
	- A national QA dashboard improved oversight and informed policy decisions.		
	- Digital registries (the e-tracker for HPV vaccination and the mUzima application for screening/treatment data) were perceived to improve data quality/timeliness and save time vs paper records.		
HPV vaccination uptake	- Significant increases in vaccination uptake	*n* = 3	(27–29)
	- SMS and automated voice calls performed equally well as vaccination reminder sub-modes (65.3% vs 63.3%, p = .86).		
	- High message-delivery reliability underpinned its results: 97.4% of intended SMS reminders were successfully delivered in the intervention arm versus 98.7% in the non-SMS arm's tracking messages.		
Cost-effectiveness / economic outcomes	- The one modeled study found app-based results communication cost-effective relative to lifetime cancer treatment costs.	*n* = 1	(42)
Post-treatment follow-up adherence	- Telephone + SMS reminders roughly quadrupled follow-up odds (aOR = 3.97, p = .016).	*n* = 1	(53)
Readiness and access indicators	- Baseline and feasibility studies provided descriptive indicators of readiness for mHealth.	*n* = 3	(41, 54, 57)
	- Mobile phone access was 91.4% in a remote forest-reserve community in Uganda and was associated with higher attendance at community outreach screening (87.3% vs 72.6% among women without access) and at health-center-based screening (96.9% vs 93.5%).		
	- Phone ownership was 98% among women attending public-sector services in South Africa, with interest in SMS screening reminders and results, but 58% had ever lost a phone or had one stolen.		
	- Caregivers of HPV-vaccine-eligible adolescents in Nigeria reported phone access alongside gaps in ownership and sharing (secondary analysis of trial baseline data).		

Screening uptake and attendance (*n* = 6) yielded the most inconsistent results (17, 19, 41, 48, 54, 56). In Nigeria, Okunade et al. (19) reported a significant SMS effect (51.0% vs. 35.7%, *p* = .031), while Addo-Lartey et al. (17) in Ghana and Linde et al. (48) in Tanzania found no significant improvement. In contrast, HPV vaccination uptake (*n* = 3) was a consistent domain, despite spanning three different countries (27–29): Uganda (27), Nigeria (28), and Kenya (29) all reported statistically significant increases in vaccination uptake with mHealth reminders. This suggests that findings in this domain are likely to generalize across national contexts. Cost-effectiveness and economic outcomes (*n* = 1) and post-treatment follow-up adherence (*n* = 1) were each reported in only a single country, Kenya (42) and Cameroon (53), respectively. These represent clear gaps in evidence rather than genuine cross-country patterns. Program and health-system implementation outcomes (*n* = 3) were confined to Nigeria, Rwanda, and Tanzania (24, 30, 50), with Ogunsola et al. (30) in Nigeria documenting concordance rising from 26% to 42% across successive implementation phases.

Five studies reported provider competency and confidence as outcomes of education and training (20, 21, 25, 50, 51). Smartphone-based mentorship was associated with high agreement between trainees and expert reviewers in Tanzania, with 96.8% overall agreement (51). Disagreement fell from approximately 10% to under 3% after the first month (51). Similarly, nurses in Ghana reported greater confidence and improved competence, described the three-month mentorship as essential for developing diagnostic and case-management skills, and valued peer-to-peer support and case discussion as additional learning methods (21). Likewise, Asgary et al. (20) found that 15 community health nurses screened 169 women after a two-week course plus three months of image-based feedback, with agreement of 98% for negative cases but 61.5% for positive cases. In the small Tanzanian study, feedback was rapid, with 52.2% of cases reviewed within five minutes and 61.2% within ten minutes (51). However, during the national scale-up, only 45.6% were reviewed within ten minutes, and 21.7% waited 24–72 h (50). In Cameroon, attendance at Project ECHO tele-mentoring sessions was associated with higher self-reported use of VIA/VILI (63.2% vs. 33.3%, *p* = .03) and greater knowledge compared to newcomers (25).

Baseline and feasibility studies (*n* = 3) (41, 54, 57) provided descriptive indicators of readiness for mHealth. Mobile phone access was high in the remote Ugandan community surveyed (91.4%), and phone access was associated with higher attendance at community outreach screening (87.3% versus 72.6% among women without access) and at health-center-based screening (96.9% versus 93.5%) (54). In South Africa, phone ownership reached 98%, and women expressed interest in receiving SMS screening reminders and results, yet 58% reported ever losing a phone or having one stolen (57). In Nigeria, a secondary analysis of trial baseline data among caregivers of HPV-vaccine-eligible adolescents reported phone access alongside gaps in ownership and sharing (41). These readiness indicators suggest that basic phone access is rarely the binding constraint, whereas the stability, sharing, and privacy of phone use determine whether SMS-based delivery can be relied upon.

### 3.6 Facilitators of mHealth interventions in cervical cancer prevention and control

Facilitators of mHealth interventions for cervical cancer prevention and control in Africa exhibited a pervasive yet graded pattern (Table 7). High mobile phone ownership and access emerged as the most frequently cited facilitator (*n* = 13) (17, 19, 28, 29, 41–43, 46, 48, 51, 54, 55, 57). Studies in Uganda (54), South Africa (57), and Kenya (43) reported widespread phone access, ownership, and comfort with SMS, indicating that basic mobile connectivity is no longer a primary constraint, even in remote settings. Low cost and cost savings were almost as widely reported (*n* = 10) (17, 19, 27, 28, 31, 42, 44, 48, 53, 57). Kitaka et al. (27) exemplified this by framing SMS/call reminders in Uganda as a low-cost, scalable alternative to the existing paper appointment card system. A Tanzanian trial cited low per-message cost and the feasibility of integrating with existing clinic lists as reasons why simple reminders were sufficient for already-motivated women (48). Real-time expert feedback, remote mentorship, and task-sharing were equally common (*n* = 10) (20, 22, 25, 30–32, 44, 47, 50, 51). In Kenya, Tonui et al. (47) reported that good cellular coverage and a median expert response time of two minutes jointly enabled real-time consultation, reducing the need for referrals from remote clinics. In Nigeria, Ogunsola et al. (30) relied on a dedicated network of nine gynecologist reviewers to sustain this mechanism at scale.

**Table 7:** Facilitators of mHealth interventions in cervical cancer prevention and control

Facilitators	Key findings	Number of studies (n)	Studies
**High mobile phone ownership and access**	- Reported ownership figures were consistently high across very different settings: 91.4% in a remote forest-reserve community in Uganda, 98% among women attending public-sector services in South Africa, and 71.3% among women attending community screening campaigns in western Kenya, where daily phone use was reported by 74.0%.	*n* = 13	(17, 19, 28, 29, 41–43, 46, 48, 51, 54, 55, 57)
	- High ownership was a facilitator and translated into high reachability during the actual intervention.		
	- Local phone penetration as a rationale for choosing an SMS-based approach over alternative delivery channels.		
**Low cost and cost savings**	- SMS-based reminder programs were repeatedly described as low-cost precisely because they avoided these conventional costs.	*n* = 10	(17, 19, 27, 28, 31, 42, 44, 48, 53, 57)
	- Telephone/SMS reminders as an inexpensive way to reach women already lost to a screening or treatment program, without requiring a physical home visit or repeat clinic appointment		
	- Smartphone- or scanner-based image transfer as a lower-cost alternative to requiring an on-site colposcopist or cytopathologist at every facility.		
	- App-supported screening and results-communication strategy is cost-effective.		
**Real-time expert feedback, remote mentorship and task-sharing**	- Rapid turnaround enabled real-time diagnostic support: median 2.0-minute expert response; feedback within 24 hours.	*n* = 10	(20, 22, 25, 30–32, 44, 47, 50, 51)
	- Enabling task-shifting to lower cadres of health workers: community health nurses or facility-level providers performing VIA independently, with remote gynecologists or oncologists serving as a second-opinion safety net rather than a required on-site presence.		
**Integration with existing systems and workflow**	- Offline modes mitigated connectivity gaps.	*n* = 10	(19, 23, 24, 30–32, 48, 50, 51, 53)
	- Integration with OpenMRS/national registries.		
	- Reminder system integration with existing General Outpatient clinic attendance patterns.		
	- Embedding smartphone-based image review directly into the routine VIA screening workflow rather than as a separate research procedure.		
**Culturally tailored / local-language content**	- Local-language preference was quantified directly in two studies: 61.2% preferred receiving SMS results in Dholuo rather than English or Swahili.	*n* = 9	(17–19, 27, 28, 42, 46, 53, 56)
	- WhatsApp audio messages delivered in English and Fante/Akan were the most favored delivery mode for educational content, preferred by 81%.		
	- Content format such as a narrative, storytelling-style video (versus a standard informational video) as a culturally resonant format for reaching Malawian women living with HIV.		
	- Cultural tailoring extended to addressing specific misconceptions rather than generic health information.		
	- Cultural and linguistic adaptation as a planned design principle intended to improve uptake and trust, framing it as a deliberate implementation strategy.		
**Patient trust, education and empowerment**	- Women reported feeling “cared for” by reminder messages.	*n* = 8	(17, 18, 21, 22, 25, 31, 46, 49)
	- Smartphone-based image review was described as building trust through transparency.		
	- Showing patients their own cervical images and explaining findings during the consultation improved patient understanding, communication with providers, and trust in the diagnosis.		
	- Privacy and a sense of decision-making autonomy among women using HPV self-sampling with WhatsApp-delivered audio education.		
	- Provider-level trust and buy-in mattered as much as patient-level trust.		
**Convenience, time-saving and reduced burden**	- Phone-based options reduced the need for repeat clinic visits and time off work.	*n* = 6	(18, 22, 43, 45, 55, 57)
	- Phone-based reminders or result delivery could reduce the need for women to miss work or pay transport costs to attend an in-person appointment.		
	- Receiving information discreetly by phone, rather than in a public clinic waiting area, was described as reducing both the practical and social burden of engaging with cervical cancer services.		
**Provision of devices/airtime/data by study team**	- Provision of smartphones and phone credit to case-finders as part of the AVIVA program's implementation model.	*n* = 4	(19, 25, 30, 51)
	- Provision of airtime to participants.		
	- Connectivity or equipment to enable their videoconferencing and image-transfer platforms, respectively.		
**Leadership/policy/institutional support**	- National partnerships underpinned scale-up.	*n* = 3	(23, 24, 50)
	- Political and leadership commitment.		
	- Aligning the study's monitoring dashboard with national cervical cancer screening program oversight (CECAP).		
**High usability and co-design**	- Co-designed provider apps were rated highly usable (23); good usability on 10/14 SUS items (32).	n = 3	(23, 24, 32)
	- Digital tools were reported to deliver a generally positive user experience and satisfaction once staff were trained (24).		

Integration of mHealth with existing systems and workflow was reported in 10 studies (19, 23, 24, 30–32, 48, 50, 51, 53). This was most concretely demonstrated in Rwanda, where Sibomana et al. (24) described the e-tracker and mUzima systems as embedded within the national OpenMRS platform, and in Nigeria, where Ogunsola et al. (30) incorporated offline functionality into the program's reviewer dashboard specifically to accommodate poor-connectivity sites. Culturally tailored interventions and local-language content were reported in 9 studies (17–19, 27, 28, 42, 46, 53, 56). Stocks et al. (46) found that 61.2% of women in western Kenya preferred results in Dholuo over English or Swahili, while in Ghana, Asare et al. (18) found that WhatsApp audio messages in Fante/Akan were preferred by 81% of participants. Patient trust, education, and empowerment (*n* = 8) clustered in Cameroon, Eswatini, Ghana, Kenya, and Tanzania (17, 18, 21, 22, 25, 31, 46, 49). In Kenya, Mungo et al. (31) found that 97% of women felt seeing their own cervical image improved their understanding. In Tanzania, improved acceptability was attributed to women feeling cared for rather than to message content itself (49).

Convenient use of mHealth and reduced burden (*n* = 6) were reported in Eswatini, Ghana, Kenya, Malawi, and South Africa (18, 22, 43, 45, 55, 57). Huchko et al. (45) found that home visits and phone-based notifications offered a lower-burden alternative to repeat clinic visits, providing direct evidence, although this was confounded by the fact that women choosing phone-based notifications already exhibited greater prior health-seeking behavior. The provision of devices or airtime by the study team (*n* = 4) was confined to Cameroon, Nigeria, and Tanzania (19, 25, 30, 51). Leadership, policy, and institutional support (*n* = 3) was reported in Kenya, Rwanda, and Tanzania (23, 24, 50), exemplified by the Ministry of Health partnership underpinning Tanzania's national screening dashboard (50). High usability or co-design of mHealth interventions was reported in Kenya, Rwanda, and Uganda (*n* = 3) (23, 24, 32).

### 3.7 Challenges to mHealth interventions in cervical cancer prevention and control

Challenges to mHealth interventions for cervical cancer prevention and control were consistently pervasive and multi-level. Sustainability, scalability, and generalizability emerged as the most frequently reported barriers (*n* = 16) (18–25, 27, 28, 30, 41, 44, 48, 51, 56) (Table 8). These issues were observed across diverse contexts, from small single-site pilot studies in Ghana (18) and Rwanda (24) to large-scale program evaluations in Nigeria (30). Notably, even large programs reported difficulty sustaining specialist reviewers’ time at scale. Studies in Uganda (27) and Malawi (56) explicitly attributed these challenges to single-site, pilot-scale designs, which limited statistical power and had not been tested in rural settings with lower connectivity. Structural health-system barriers, including geographical distance, staffing shortages, and resource constraints, were similarly widespread (*n* = 14) (17, 19, 21, 24, 28–30, 32, 42–44, 47, 50, 51), reported from Ghana to Uganda. For instance, in the Kenyan study, Tonui et al. (47) found that clinics were located 72–150 km from the nearest tertiary referral center. In Rwanda, Sibomana et al. (24) reported that health workers faced equipment shortages, unreliable internet access, and a need for increased staff and improved system interoperability to sustain adoption.

**Table 8:** Challenges to mHealth interventions in cervical cancer prevention and control

Challenges	Key findings	Number of studies (n)	Studies
Sustainability, scale-up and generalizability concerns	- Sustaining specialist reviewer time and scaling mentorship capacity across many primary health centers as an ongoing concern despite the program's demonstrated success	*n* = 16	(18–25, 27, 28, 30, 41, 44, 48, 51, 56)
	- Predominantly single-site pilots with small samples limiting generalizability.		
Structural and health-system barriers (distance, staff, resources, wait times)	- Distance to specialist or treatment facilities was a recurring structural constraint even in technologically successful studies.	*n* = 14	(17, 19, 21, 24, 28–30, 32, 42–44, 47, 50, 51)
	- Limited staff time.		
	- Competing clinical duties.		
	- Resource and equipment gaps affected image-based diagnostic studies.		
Network and connectivity issues	- Having problems connecting to or maintaining a connection during tele-mentoring sessions.	*n* = 12	(24, 25, 27, 30–32, 44, 47, 50, 51, 53, 55)
	- Connectivity issues at times caused lost or delayed image uploads.		
	- Connectivity gaps as a reason offline functionality had to be built into their respective systems.		
	- Cellular network coverage was less consistent in rural and remote areas.		
Cost, financial burden (to users or system)	- Data costs and phone bills remained a barrier despite low per-message costs of the interventions themselves.	*n* = 6	(17, 19, 32, 43, 50, 52)
	- Costs to the health system were raised in large program evaluations.		
	- Transport or missed-work costs could outweigh the convenience benefit of a phone-based reminder.		
	- Sustainability of the smartphone telemedicine system — beyond the research funding period — remained an open question raised by health workers.		
Diagnostic and test performance limitations	- Low CIN2+ sensitivity (21.4–35.7%) and modest PPV (63.5%) suggest residual over/under-diagnosis risk.	*n* = 6	(22, 30, 31, 44, 47, 52)
	- Subjectivity of the underlying visual test.		
Phone sharing, ownership gaps, loss and theft	- 58% of women reported ever losing or having a phone stolen.	*n* = 6	(29, 41, 46, 53, 55, 57)
	- Ownership instability as an equity concern.		
	- Raising continuity-of-contact concerns.		
	- Unreachable phone numbers.		
	- Phone-sharing—where a woman's phone is also used by a spouse, family member, or other household member.		
Privacy and confidentiality concerns	- Concerns centered on others seeing SMS results and discomfort receiving abnormal results by text.	*n* = 4	(32, 46, 55, 57)
	- Confidentiality of HPV results delivered by phone as a concern specifically in the context of phone-sharing.		
	- Confidentiality of images and clinical data transmitted through the smartphone telemedicine system as a concern requiring clear data-governance safeguards.		
	- Cultural sensitivity of cervical cancer and gynecological care heightened the risk of unintended disclosure when phones were shared with partners or household members.		
Low literacy and language barriers	- Low information literacy.	*n* = 4	(24, 29, 55, 57)
	- Mismatch between the language of intervention content and participants’ preferred or best-understood language.		
	- Limited computer literacy among some health workers as a barrier to confident use of the digital registry systems.		
Technical and image-quality issues	- Poor lighting and positioning during image capture contributed to images being rated lower quality.	*n* = 4	(25, 30, 31, 50)
	- Technical difficulties operating the teleconferencing equipment.		
	- Issues in maintaining consistent smartphone hardware and camera quality across a national-scale program.		
Stigma and fear	- Fear of a positive diagnosis, or of the treatment procedure, as a barrier discouraging women from responding to reminders about treatment or follow-up.	*n* = 4	(17, 21, 43, 53)
	- Lingering community-level stigma around cervical cancer and gynecological examination as a barrier to women's willingness to be screened in the first place.		
Loss to follow-up and attrition	- Loss to follow-up and participant withdrawal.	*n* = 2	(19, 48)

Network and connectivity failures were reported almost as frequently (*n* = 12) and exhibited the most even geographical distribution (24, 25, 27, 30–32, 44, 47, 50, 51, 53, 55). These barriers spanned Cameroon (25), where 56.3% of tele-mentoring participants reported connection problems, Tanzania (50), Uganda (32), and Nigeria (30), where unreliable rural internet connectivity increased workload for imaging and uploading. This indicates that connectivity is a near-universal constraint across the region's diverse infrastructure, rather than a localized issue. In contrast, some challenges demonstrated clearer geographic clustering. Diagnostic and test-performance limitations, such as low CIN2+ sensitivity in Kenya (31) and modest positive predictive value in Eswatini (22), were concentrated in countries implementing image-based or AI-supported diagnostic programs (Cameroon, Eswatini, Kenya, Nigeria), as opposed to those utilizing SMS-only interventions.

Phone ownership, sharing, and access gaps (*n* = 6) extended beyond adult women to adolescent populations (29, 41, 46, 53, 55, 57). In Kenya, Onyango et al. (29) found that adolescent girls often lacked personal phones, necessitating guardian mediation for HPV vaccination reminders. In Cameroon, Nkfusai et al. (53) linked persistently high rates of missed follow-up to limited phone access among low-income groups and poor cellular coverage in remote areas. Privacy and confidentiality concerns clustered in Kenya, Malawi, South Africa, and Uganda. Moodley et al. (57) in South Africa and Chapola et al. (55) in Malawi both associated discomfort with phone-delivered results with documented phone-sharing patterns in these countries. For example, 58% of women reported phone loss or theft in Moodley et al. (57).

The cultural sensitivity of cervical cancer and gynecological care heightens these privacy risks, especially where phones are shared. Partners, relatives, or other household members using the same handset may see or overhear SMS results, reminders, calls, or cervical images, so a message about screening, HPV status, or treatment can unintentionally disclose intimate health information. Included studies reported concerns about others seeing SMS results, discomfort receiving abnormal results by text, and the confidentiality of cervical images and clinical data in smartphone telemedicine, which called for clear data-governance safeguards (32, 46, 55, 57). The discretion that made phone-based delivery attractive, because women could receive information without waiting in a public clinic (18, 22, 43, 45, 55, 57), therefore depended on private access to a handset. Stigma and persistent misconceptions further heighten this sensitivity in African settings. In Ghana, some women in the intervention arm still held dietary misconceptions about the causes of cervical cancer despite culturally tailored messaging (17), and in Kenya, Choi et al. (43) concluded that SMS alone was insufficient to overcome structural barriers such as stigma and low-risk perception.

Cost and financial burden (*n* = 6) affected both users and health systems and were reported across West, East, and Central Africa (17, 19, 32, 43, 50, 52). Although the per-message cost of SMS interventions was low, data costs and phone bills remained a barrier for some women. Transport and missed-work costs could also outweigh the convenience of a phone-based reminder, and larger program evaluations could raise costs for the health system. Health workers questioned whether the smartphone telemedicine system could be sustained beyond the research funding period. These findings suggest that low-cost delivery does not remove the financial barriers women face in accessing services, nor does it address the funding needed to sustain digital tools at scale. Loss to follow-up and participant attrition were the least reported challenges (*n* = 2), confined to Nigeria (19) and Tanzania (48).

## 4 Discussion

This scoping review indicates that mHealth is a promising approach to cervical cancer prevention and control in Africa, particularly image-based diagnosis with tele-expert review and SMS/audio messaging, although effectiveness varied by intervention type and outcome. Interventions disproportionately focused on screening within the care continuum, with few studies examining the role of mHealth in primary prevention and post-treatment follow-up (27–29, 53). Similarly, recent reviews of the cervical cancer intervention literature reveal a predominant focus on prevention and screening. For instance, Rivero-Mendoza et al. (58) delineated health system strategies to enhance screening uptake in sub-Saharan Africa, while Atnafu et al. (59) synthesized multilevel factors influencing prevention, screening, early detection, and management. Cumulatively, these reviews suggest that evidence for post-treatment and survivorship-oriented interventions is considerably less prominent than that for screening-focused initiatives (58, 59).

Studies on mHealth and cervical cancer telemedicine largely emphasize screening and diagnostic support (6, 60). Image-based diagnosis with tele-expert review emerged as one of the most common mHealth approaches (20–22, 30–32, 44, 47, 50–52). Tendobi et al. (33) applied a similar model, in which non-specialist providers captured smartphone cervical images for remote specialist confirmation; expert review of the first 100 cases also served as training, mirroring the mentorship function described by Asgary et al. (20, 21) and Yeates et al. (51). A shift toward automation was also evident: Holmström et al. (44) used deep-learning interpretation, and Parham et al. (34) and Hu et al. (35) replaced human reviewers with automated visual evaluation, which may reduce reliance on scarce specialists. Basic phones and smartphones were the dominant devices, consistent with the observation by Tendobi et al. (33) that they are widely available; however, Parham et al. (34) and Hu et al. (35) found that algorithm performance was device-specific. Diagnostic accuracy also varied. Mungo et al. (31) reported low sensitivity among off-site colposcopists (21.4–35.7%), whereas Tendobi et al. (33) reported that for high-grade lesions, the sensitivity was 88.9% and the negative predictive value was 99.7%; for any lesion, the sensitivity was 66.0% and the negative predictive value was 95.3%. The evidence for telemedicine in gynecologic oncology is still emerging and includes few rigorous studies on remote survivorship and follow-up models (60, 61).

The stronger effect of message reminders on HPV vaccination uptake (27–29), compared with the variable results for screening uptake and treatment linkage (30, 42, 43, 45, 46, 50, 52, 55, 57), is consistent with extra-regional trial evidence: Daly et al. (62) demonstrated that behaviorally informed SMS reminders significantly increased HPV vaccine uptake in Georgia (ORs 1.34–1.65), extending positive signals from African trials such as Okunade et al. (28). A study of an mHealth intervention to increase adherence to triage among HPV-positive women, conducted in Argentina by Paolino et al. (15), found that the acceptability of SMS messages was high (97%) among women who had received at least one message, and among them, 76% attended a health center to retrieve their results. Of those who retrieved their results, 90% reported that the SMS message had influenced their decision to attend.

Conversely, the message-based effects on screening and follow-through align with findings from the United States and India. Ciceron et al. (63) noted that while a text-messaging intervention was feasible and acceptable for cervical cancer prevention among women with HIV in Washington, DC, scalable behavior change likely requires pairing it with in-person reinforcement. Similarly, Hombaiah et al. (16) reported that a mobile health application in Mysuru, India, increased cervical cancer knowledge but yielded only a modest, approximately 5% increase in Pap-test uptake. This knowledge-behavior gap, evident in null-result trials from Ghana (17) and Tanzania (48), appears to be a cross-contextual phenomenon.

The predominant facilitating factors included high mobile phone ownership, low intervention costs, and real-time expert mentorship (43, 54, 57). However, the assumption of universal phone access requires qualification. The GSMA's 2026 Mobile Gender Gap Report estimated that in low- and middle-income countries (LMICs), 84% of women own a mobile phone, 64% own a smartphone, and 65% use mobile internet (64). In Argentina, Paolino et al. (15) reported that a substantial majority of participants (88%) possessed a mobile phone with internet access, and 89% reported using social networks. A small minority (13%) shared their phone with family members. Despite these figures, disparities in mobile access and usage persist. Women in LMICs remain less likely than men to access mobile phones, mobile money, mobile internet, and related services (64). These gaps are especially pronounced among the most underserved women, including those with low literacy, low income, living in rural areas, or having a disability (64). This tempers optimism about ownership-based facilitators and is corroborated by Stocks et al. (46), who found that although mobile phone ownership was high in western Kenya, it was often shared or intermittent, affecting the reliability of follow-up. The salience of culturally and linguistically tailored content, evidenced by Asare et al. (18) and Stocks et al. (46), was independently affirmed by research from Bonful et al. (65), which showed that culturally tailored SMS content should address women's limited knowledge of screening, fear, time constraints, and cost-related concerns, while incorporating information delivered or reinforced by trusted health professionals.

Privacy and cultural sensitivity cut across all intervention types. Cervical screening, HPV testing, and gynecological examination are intimate and often stigmatized, and women frequently share phones with partners or other household members. SMS reminders, results, calls, and cervical images can therefore be read, overheard, or seen by others, leading to unintended disclosure and household scrutiny. The included studies raised these concerns for SMS-delivered results and for images transmitted through telemedicine systems (32, 46, 55, 57). This may help explain why one-way messaging alone produces inconsistent effects on screening uptake. Programs should therefore ensure informed choice and confidentiality and respect women's autonomy (66). An optimal notification strategy would pair the health information and personal support of a face-to-face visit from a trusted provider with the efficiency and privacy of phone-based notification (45). This could be achieved through better-scripted phone calls, more face-to-face time with community health volunteers (CHVs) at the time of screening, or improved counseling materials given to patients after testing (45).

Sustainability, structural health-system barriers, and connectivity emerged as pervasive challenges, echoing findings in the broader sub-Saharan African literature (58, 59). Provider-side readiness is a critical, often underexamined precondition. In sub-Saharan Africa, Delie et al. (67) found that pooled proportions of good cervical cancer screening knowledge and positive attitudes among healthcare providers were only 67.9% and 55.3%, respectively. This, coupled with the caution expressed by Mutebi et al. (68), suggests that workforce knowledge and system readiness, rather than technology access alone, may be the binding constraint on mHealth effectiveness.

This review highlights practical, policy, and research implications for cervical cancer control in Africa. Reminder-based mHealth interventions showed favorable results for HPV vaccination uptake across the included studies, suggesting they may hold promise for primary prevention. However, the variable impact of one-way SMS on screening participation suggests that message-based tools should be combined with interactive or human-mediated elements, such as tele-expert review or phone follow-ups, rather than used alone. The focus of mHealth investments on screening, while neglecting post-treatment follow-up and primary prevention, underscores the need for national cervical cancer strategies to fund digital tools across all stages of care. The ongoing structural barriers to technological acceptance highlight the importance of infrastructure investment rather than further acceptability testing as a policy priority. Additionally, the feasibility of task-shifted, tele-mentored diagnostic models has implications for workforce planning in countries with limited specialists and should be tested in broader contexts.

## 5 Limitations and recommendations

This review has some limitations at both the primary-study and review levels. Few studies employed a randomized design, and small sample sizes often precluded comparative effectiveness assessment. Several trials were identified as potentially biased due to loss to follow-up or confounding factors. At the review level, most evidence comes from East African countries, particularly Kenya, which limits the applicability of the findings. In this review, only four bibliographic databases (CINAHL, MEDLINE, Scopus, and Web of Science) were searched, supplemented by Google and Google Scholar; therefore, relevant studies indexed elsewhere may have been missed. Screening was limited to the first 100 results in Google Scholar and the first 10 pages in Google, which restricts completeness and reproducibility. The search term “Africa” could not be expanded in Scopus and Web of Science; thus, records naming only a specific country or city may have been missed. Future reviews should also consider testing a validated geographic search filter, such as that developed by Pienaar et al. (39), which combines country- and region-level terms to identify African randomized controlled trials in MEDLINE and EMBASE; however, as that filter was developed and validated for RCTs only and improved precision at some cost to sensitivity (73%–74%), its performance for the broader range of study designs and databases used in this review, including CINAHL, Scopus, and Web of Science, would need to be independently assessed before adoption.

Combining the condition (S2) and prevention (S3) blocks with AND may have excluded records that used a prevention/screening term without cervical-cancer-specific terminology. Restricting the review to English-language publications from 2016 to 2026 may also have excluded relevant studies from other languages, particularly francophone literature. Future reviews should search additional databases, include country names alongside regional terms, and consider searching in other languages, such as French, Portuguese, and Spanish, to improve coverage.

The intervention type, outcome domain, and classification of effectiveness required interpretative coding because of the absence of a standardized taxonomy. As a scoping review, no formal risk-of-bias assessment or effect-size pooling was performed. This review recommends that mHealth implementers focus on interactive or human-mediated mHealth strategies (such as tele-expert review and calls combined with SMS reminders) rather than relying on standalone one-way messages, which have shown inconsistent effectiveness. National programs should broaden investment in underrepresented areas of HPV vaccination and post-treatment follow-up, ensuring scale-up is paired with improved connectivity and explicit attention to the gendered digital divide and to confidentiality safeguards for women who share phones. For future research, larger multi-country randomized trials with follow-up beyond the usual two to six months, routine cost-effectiveness reporting, and deliberate expansion into regions outside East Africa are necessary to build a stronger evidence base to inform future cervical cancer elimination policies. Furthermore, no stakeholder consultation exercise was undertaken. Although Arksey and O'Malley (36) proposed consultation as an optional sixth stage of the scoping review framework, and Levac et al. (37) recommended it as an integral component, this review drew on the published literature alone. As a result, the findings have not been validated or contextualized by the perspectives of those who design, implement, or use mHealth interventions for cervical cancer screening. Future updates of this review would benefit from consulting relevant stakeholders, such as cervical cancer screening program managers, mHealth implementers, and women with lived experience of screening, to validate, contextualize, and extend these findings.

## 6 Conclusion

This scoping review analyzed data from 32 studies on mHealth strategies for cervical cancer prevention and control across Africa. mHealth interventions included smartphone-based image capture and tele-expert review, along with SMS or audio messaging, which together accounted for over half of the evidence. These strategies mostly focused on screening, while primary prevention and post-treatment follow-up received less attention, despite the former showing some of the most significant and consistent results. Outcomes included diagnostic accuracy, feasibility, treatment initiation, and HPV vaccination, with vaccination reminders demonstrating a consistently positive impact. Factors such as high mobile phone ownership, low costs, and real-time expert guidance facilitated implementation, whereas challenges like sustainability, health system barriers, and connectivity issues were common. These reflect infrastructural rather than attitudinal barriers in resource-limited settings. Overall, the review highlights mHealth's substantial potential to improve cervical cancer control in Africa, especially in screening and HPV vaccination. Achieving this on a national scale, however, requires targeted investments in overlooked stages of care, infrastructure development, and more comprehensive multi-country research beyond initial pilot studies.

## Data Availability

The original contributions presented in the study are included in the article/Supplementary Material. Further inquiries can be directed to the corresponding author.
